# Association of methylenetetrahydrofolate reductase gene (C677T) with the risk of hypertension in Morocco

**DOI:** 10.1186/s13104-015-1772-x

**Published:** 2015-12-12

**Authors:** Sanaa Nassereddine, Yaya Kassogue, Farah Korchi, Rachida Habbal, Sellama Nadifi

**Affiliations:** Genetics and Molecular Pathology Laboratory, Medical School of Casablanca, University Hassan II, 19 Rue Tarik Ibnou Ziad, BP. 9154 Casablanca, Morocco; Department of Cardiology, Ibn Rochd University Hospital, Casablanca, Morocco

**Keywords:** Hypertension, Susceptibility, MTHFR C677T polymorphism

## Abstract

**Background:**

Hypertension is a multifactorial disease caused by the interaction between genetic and environmental factors. Mutations in the methylenetetrahydrofolate reductase gene (MTHFR) have been known to be associated with the risk of cardiovascular disease as well as hypertension. This case–control study was conducted out to measure the association of the polymorphism C677T of MTHFR with the risk of hypertension.

**Methods:**

Polymerase chain reaction followed by restriction fragment analysis length was used to identify MTHFR C677T genotypes in patients 101 patients and 102 age and sex matched healthy controls. Odds ratio with 95 % confidence interval was used to assess the risk of association.

**Results:**

The distribution of demographic and clinical features of patients showed no particular trend (p > 0.05). However, the frequency of homozygous 677T allele was higher in patients with a family history of heart disease (30.4 vs. 9 %, p = 0.031). Interestingly, the mutant 677TT genotype was significantly associated with the susceptibility of hypertension when compared to the wild type 677CC genotype (OR 5.4, CI 1.4–19.8, p = 0.008). In addition, the recessive model 677TT vs. 677CC/CT was found to be associated with the risk of hypertension (OR 5.3, CI 1.5–19.1, p = 0.005). However, the dominant model was not associated with the risk of hypertension in our cohort (OR 1.3, CI 0.7–2.2, p = 0.4).

**Conclusions:**

Based on our findings, the homozygous mutant for 677TT of MTHFR gene is associated with the risk of hypertension in our population. Further studies with larger sample sizes are needed to confirm the results of this study.

## Background

Hypertension is a major public health concern in the world. It is found to be the main factor in the occurrence of myocardial infarction, stroke, cardiac and renal failure and later lesions of the retina of the eyes [1, 2]. In Morocco, the prevalence of hypertension in men and women is 30.2 and 37 %, respectively, with an overall prevalence of 33.6 % [3, 4]. The etiology of hypertension, like most of multifactorial diseases is not known. However, the pathogenesis of hypertension can be explained by the interaction between many factors such as sodium intake, overweight, alcohol, smoke and the genetic background of subjects [5, 6]. Many authors have shown the fundamental role of genetic factors not only in the development of hypertension, but also in the individualization of treatment [7, 8]. Methylenetetrahydrofolate reductase gene (MTHFR) is particularly involved in the metabolism of homocysteine and folate by catalyzing the 5, 10-methylenetetrahydrofolate into 5-methylenetetrahydrofolate. The MTHFR gene has been mapped to chromosome 1p36.3 and encodes an enzyme composed of 656 amino acids [9]. It was reported that, conditions such as hyperhomocysteinemia and homocystinuria are associated with mutations in the MTHFR gene. The single nucleotide polymorphism 677C > T (Alanine222Valine) of MTHFR reduces the enzymatic activity [10]. As a result, individuals this mutation are exposed to a high level of homocysteine in plasma and are more likely to develop cardiovascular disease, due to the absence of conversion of homocysteine to methionine by 5-methylenetetrahydrofolate [11, 12]. It has been shown that the MTHFR enzyme activity decrease is attributable to the loss of cofactor of Riboflavin. Thus, the supplementation of this latter, contributes to the reduction of hyperhomocysteinemia in hypertensive patients with the variant of MTHFR [13–15]. A recent study showed that supplementation with Riboflavin in hypertensive patients carrying the homozygous genotype mutant 677TT of MTHFR, without other cardiovascular diseases, has considerably improved the value of systolic blood pressure, paving the way for personalized medicine [16]. It is worth noting, that numerous studies investigated the relationship between the C677T polymorphism of MTHFR and many diseases, including breast cancer [17, 18], colorectal cancer [19], ischemic stroke [20, 21], inflammatory bowel disease [22] and hypertension [7, 23, 24]. In the light of the limited data on the variants of the MTHFR and hypertension in our population, and the role of vitamin B2 in the improvement of blood pressure in hypertensive patients with a mutant variant of the MTHFR gene, this case–control study was carried out to investigate the association of 677C > T polymorphism of MTHFR with the risk of hypertension in a sample of the Moroccan population.

## Methods

### Study population

The study included 101 outpatients (77 females and 24 males) with a mean age of 61.6 ± 9 (range 40–87 years) and 102 age and sex matched unrelated healthy control subjects with a mean age of 59.24 ± 10.7 (range 40–87 years). Patients have been confirmed with hypertension by cardiologists according to the criteria of International Society of Hypertension [systolic blood pressure (SBP) ≥ 140 mmHg or diastolic blood pressure (DBP) ≥ 90 mmHg] [25]. Patients were recruited from the department of cardiology of the Ibn Rochd University Hospital in Casablanca, Morocco from 2011 to 2013. The control group was normotensive after medical examination (SBP < 140 mmHg or DBP < 90 mmHg) without any family history of cardiovascular, diabetes and thyroid diseases and was recruited at the laboratory of genetic and molecular diseases, Faculty of Medicine, the Hassan II University in Casablanca. The control subjects were composed of employees working at the faculty of medicine. The study received the approval from the local Ethics Committee and all participants accepted and signed the informed consent. Five milliliters of venous blood were collected from each participant in an EDTA tube for the genotyping of the MTHFR gene.

### Identification of MTHFR C677T polymorphism

Genomic DNA was extracted from white blood cells by using the salting-out method described previously by Miller et al. [26]. Polymerase chain reaction followed by the restriction fragment length polymorphism was used to genotype the C677T polymorphism of MTHFR. The conditions of amplification and digestion have been well documented previously in our laboratory [19]. The digested PCR products after separation on a 3 % agarose gel, stained with ethidium bromide, showed one band of 198 bp corresponding to the wild type homozygous (CC), three bands of 198, 175 and 23 bp for the heterozygous (CT), two bands of 175 and 23 bp for the mutated homozygous (TT).

### Statistical analysis

The statistical package for the social sciences SPSS version 16 (SPSS Inc., Chicago, IL, USA) was used to perform all statistical analyses. The difference in the distribution of demographic data, clinical characteristics of patients as well as their family history and MTHFR C677T genotypes were conducted by Fisher’s exact test. Hardy–Weinberg equilibrium in the cases and controls was evaluated by Chi square test. The estimation of the association between MTHFR C677T profiles and the risk of hypertension was evaluated by Fisher’s exact test followed by the calculation of an odds ratio (OR) with 95 % confidence interval. Statistical tests were considered significant when the p value was less than 0.05.

## Results

The present case–control study assessed the distribution of the C677T polymorphism of MTHFR in 101 patients with hypertension and 102 controls without any history of hypertension. The frequencies of alleles and genotypes of C677T in patients and controls did not deviate from Hardy–Weinberg equilibrium (p > 0.05). As shown in Table 1, the distribution of CC, CT and TT genotypes in patients with respect to demographic data and clinical features (gender, age, body mass index, diabetes, dyslipidemia, risk stage, smoking habit) did not show a particular trend (p > 0.05). The stratification of patients according to their family history showed that carriers of mutated homozygous TT with a family history of heart disease were significantly associated with the risk of hypertension (30.4 vs. 9 %, p = 0.031). The distribution of other family histories such as hypertension, stroke, heart disease, diabetes, kidney disease and dyslipidemia were comparable between the different genotypes of MTHFR C677T with p > 0.05 (Table 2). In Table 3, the frequency of the homozygous wild type CC was higher in the control group than in patients (52.9 vs. 46.5 %). However, the frequency of the mutant TT was higher in cases than controls (13.9 vs. 2.9 %) and the TT genotype was found to be statistically associated with the risk of hypertension when compared with carriers of homozygous wild type CC (OR 5.4, CI 1.4–19.8, p = 0.008). Furthermore, the comparison of TT to CC/CT carriers in the recessive model showed the same trends with a risk of 5.3 (p = 0.005). However, the dominant model did not show any particular effect concerning the risk of hypertension. The frequency of mutant 677T allele was higher in cases compared to controls (34 vs. 25 %), but not significantly.

**Table 1:** Distribution of clinical and demographic features of the patients, according to the different genotypes of MTHFR C677T

Parameters	C677T N (%)			χ^2^	p
	CC	CT	TT		
Gender					
Female	35 (45.5)	30 (39)	12 (15.5)	0.8	0.6
Male	12 (50)	10 (41.7)	2 (8.3)		
Age (years)					
40–60	27 (49.1)	20 (36.4)	8 (14.5)	0.5	0.7
61–87	20 (43.5)	20 (43.5)	6 (13)		
BMI (kg/m^2^)					
18–24	14 (48.3)	9 (31)	6 (20.7)	5.2	0.3
25–30	19 (43.2)	22 (50)	3 (6.8)		
>30	14 (50)	9 (32.1)	5 (17.9)		
Diabetes					
Yes	14 (45.2)	12 (38.7)	5 (16.1)	0.2	0.9
No	33 (47.1)	28 (40)	9 (12.9)		
Dyslipidemia					
Yes	15 (41.7)	14 (38.9)	7 (19.4)	0.5	0.5
No	32 (49.2)	26 (40)	7 (10.8)		
Stage of risk					
Low	9 (34.6)	12 (46.2)	5 (19.2)	6.35	0.2
Medium	24 (58.5)	11 (26.8)	6 (14.6)		
High	14 (41.2)	17 (50)	3 (8.8)		
Smoking habit					
Yes	6 (46.2)	5 (38.5)	2 (15.4)	0.03	1
No	41 (46.6)	35 (39.8)	12 (13.6)		

*FH* family history, *CC* homozygous wild type, *CT* heterozygous, *TT* homozygous variant

**Table 2:** Distribution of MTHFR genotype according to patient’s family history

Family history	C677T N (%)			χ^2^	p
	CC	CT	TT		
Hypertension					
Yes	29 (50)	23 (39.7)	6 (10.3)	1.6	0.5
No	18 (41.9)	17 (39.7)	8 (18.6)		
Heart disease					
Yes	8 (34.8)	8 (34.8)	7 (30.4)	6.9	0.031
No	39 (50)	32 (41)	7 (9)		
Stroke					
Yes	7 (43.8)	7 (43.8)	2 (12.5)	0.14	0.9
No	40 (47.1)	33 (38.8)	12 (14.1)		
Diabetes					
Yes	18 (40)	20 (44.4)	7 (15.6)	1.4	0.5
No	29 (51.8)	20 (35.7)	7 (12.5)		
Kidney disease					
Yes	1 (12.5)	6 (75)	1 (12.5)	4.9	0.08
No	46 (49.5)	34 (36.6)	13 (14)		
Dyslipidemia					
Yes	12 (48)	8 (32)	5 (20)	1.4	0.5
No	35 (46.1)	32 (42.1)	9 (11.8)		

*CC* homozygous wild type, *CT* heterozygous, *TT* homozygous variant

**Table 3:** Distribution of **g**enotypes and alleles of MTHFR in patients and controls

	Patients N (%)	Control N (%)	OR (95 % CI)	p
C677T				
CC	47 (46.5)	54 (52.9)	Ref.	
CT	40 (39.6)	45 (44.2)	1 (0.6–1.8)	1
TT	14 (13.9)	3 (2.9)	5.4 (1.4–19.8)	0.008
CC/CT^a^	87 (86)	99 (97)	Ref.	
TT	14 (14)	3 (3)	5.3 (1.5–19.1)	0.005
CC^b^	47 (46)	54 (53)	Ref.	
CT/TT	54 (54)	48 (47)	1.3 (0.7–2.2)	0.4
C	134 (66)	153 (75)	Ref.	
T	68 (34)	51 (25)	1.5 (1–2.3)	0.06
HWE: p value	0.27	0.11		

*HWE* Hardy–Weinberg Equilibrium, *N* number

^a^Recessive model

^b^Dominant model

## Discussion

Hypertension is a multifactorial disease that involves both genetic and environmental factors and it is known to be a common risk factor for kidney and heart complications. Hereditary genetic differences in MTHFR gene and the risk of hypertension have been discussed by many authors with conflicting results. In the current study, the genotype correlation of patients with regard to parameters such as gender, age, body mass index, diabetes, dyslipidemia, the stage of risk and smoking showed no significant trend. Similar results were reported by Gay et al. in the Chinese population [27]. The frequency of the homozygous mutant 677TT was statistically higher in patients with a history of heart disease in the family. This finding might help in the genetic counseling. However, other family histories showed a comparable distribution among the different genotypes of MTHFR C677T. However, Alghasham et al. in Saudi Arabia showed no association of the patient’s family history and MTHFR C677T genotypes [28]. We found that, the frequency of the wild type homozygous CC was higher in controls than in patients and subjects carriers of 677TT were associated with an increased risk hypertension (OR 5.4, p = 0.008). In addition, in the recessive model, we noticed that patients with the 677TT genotype were significantly associated with the risk of hypertension (OR 5.3, p = 0.005). Markan et al. reported similar observations in the Indian population [29]. Yan et al. also reported in a meta-analysis a strong association of MTHFR C677T with the risk of hypertension among Asians, Caucasians and Chinese subjects [30]. Similar findings were obtained in the population of Turkey [7]. Many authors have reported that the MTHFR C677T polymorphism has been found to be an independent factor of hypertension in different ethnic groups [31–33] as well as of severe diastolic hypertension in pregnant women [34]. These above studies showed that the MTHFR C677T polymorphism is associated with a high level of homocysteine. This study did not assess the level of homocysteine. However, a previous study conducted by Bennouar et al. in 2007 showed that the 677TT genotype was statistically higher in subjects with coronary artery disease and was found to be associated with hyperhomocysteinemia in Morocco [35]. In this study, it should be noted some limitations. First, our sample size was small, which reduces the statistical power. Secondly, the absence of the determination of homocysteine in patients and controls failed to establish the correlation between the variants of MTHFR and the values of homocysteine. Thirdly, hypertension is a complex disease; other candidate genes may contribute to the susceptibility of hypertension. With respect to these limitations, the results of this study should be interpreted with caution. Other studies of large sample, overcoming the current limitations might be interesting in the understanding of the role of candidate genes in the susceptibility of hypertension in our population.

## Conclusion

We have looked the influence of MTHFR C677T and the risk of hypertension in a sample of the Moroccan population. We have shown that, the homozygous mutant 677TT was significantly associated with the risk of hypertension. Additionally, the recessive model showed the same trend. Considering the size of our sample, further studies will be needed to confirm our findings.
